# Exploiting the metabolic dependencies of the broad amino acid transporter SLC6A14

**DOI:** 10.18632/oncotarget.27758

**Published:** 2020-12-01

**Authors:** Francesca R. Dejure, Joachim Butzer, Ralph K. Lindemann, Balca R. Mardin

**Affiliations:** ^1^BioMed X Institute (GmbH), Heidelberg, Germany; ^2^Translational Innovation Platform Oncology, Merck KGaA, Darmstadt, Germany

**Keywords:** SLC6A14, metabolic stress, transcriptional regulation, methionine, AMPK

## Abstract

Tumor cells typically enhance their metabolic capacity to sustain their higher rate of growth and proliferation. One way to elevate the nutrient intake into cancer cells is to increase the expression of genes encoding amino acid transporters, which may represent targetable vulnerabilities. Here, we study the regulation and function of the broad amino acid transporter SLC6A14 in combination with metabolic stress, providing insights into an uncharacterized aspect of the transporter activity. We analyze the pattern of transcriptional changes in a panel of breast cancer cell lines upon metabolic stress and found that SLC6A14 expression levels are increased in the absence of methionine. Methionine deprivation, which can be achieved via modulation of dietary methionine intake in tumor cells, in turn leads to a heightened activation of the AMP-activated kinase (AMPK) in SLC6A14-deficient cells. While SLC6A14 genetic deficiency does not have a major impact on cell proliferation, combined depletion of AMPK and SLC6A14 leads to an increase in apoptosis upon methionine starvation, suggesting that combined targeting of SLC6A14 and AMPK can be exploited as a therapeutic approach to starve tumor cells.

## INTRODUCTION

One of the hallmarks of tumors is their deregulated metabolism. Cancer cells typically have a high demand for nutrients such as glucose and amino acids [1]. Since amino acids are used for the synthesis of macromolecules required for sustaining the accelerated growth of tumor cells, blocking the amino acid transporters may present as a viable therapeutic option, leading to amino acid starvation selectively in tumor cells [2]. Consistent with the idea that the function of the amino acid transporters can be more critical for the maintenance of tumor cells, several amino acid transporters are reported to be overexpressed in a wide spectrum of tumors [3, 4]. For instance, in breast cancer, the metabolism of non-essential amino acids is found to be altered [5] and the expression of amino acid transporters correlates with tumor growth and progression [6–8].

Amino acid transporters belong to the solute carrier (SLC) family whose members are able to mediate the transport of a plethora of molecules across cellular membranes. To date, approximately 50 amino acid transporters have been identified and distinguished based on their subcellular localization (plasma membrane, mitochondria, lysosomes), their substrate specificity and their mechanism of transport [9].

The amino acid transporter SLC6A14 (ATB^0,+^) is a highly concentrative symporter which makes use of the sodium and chloride gradient to uptake amino acids. With the exception of the polar negatively charged amino acids, aspartate and glutamate, SLC6A14 is able to mediate the uptake of all proteinogenic amino acids. Although *in vitro* studies have revealed variable affinities of each substrate, SLC6A14 can transport the highest range of amino acids compared to all the other amino acid transporters [10]. In addition, while it is expressed at low levels in normal tissues and SLC6A14 KO mice are viable [11], SLC6A14 has been reported to be upregulated in breast, colon, lung and pancreatic tumors [12]. Due to its unique properties, SLC6A14 emerges as a relevant target for cancer therapy. However, under which conditions blocking SLC6A14 may lead to selective killing of tumor cells has not yet been explored.

In this study, we examined the potential of SLC6A14 as a therapeutic target in breast tumors. In particular, we focused on the evaluation of SLC6A14 function under conditions of metabolic stress which are generally experienced by tumor cells either because of intrinsic characteristics of the tumor microenvironment or as a result of drugs targeting metabolic pathways. We characterized the pattern of transcriptional changes upon stress which highlights the high dynamic regulation of amino acid transporter genes. We found that inhibition of SLC6A14 becomes particularly relevant under metabolic stress and results in the activation of AMP-activated kinase (AMPK). We propose that the combined targeting of SLC6A14 and AMPK can be exploited as a therapeutic approach to starve tumor cells.

## RESULTS

### SLC6A14 is heterogeneously expressed in breast cancer

**Figure 1:**
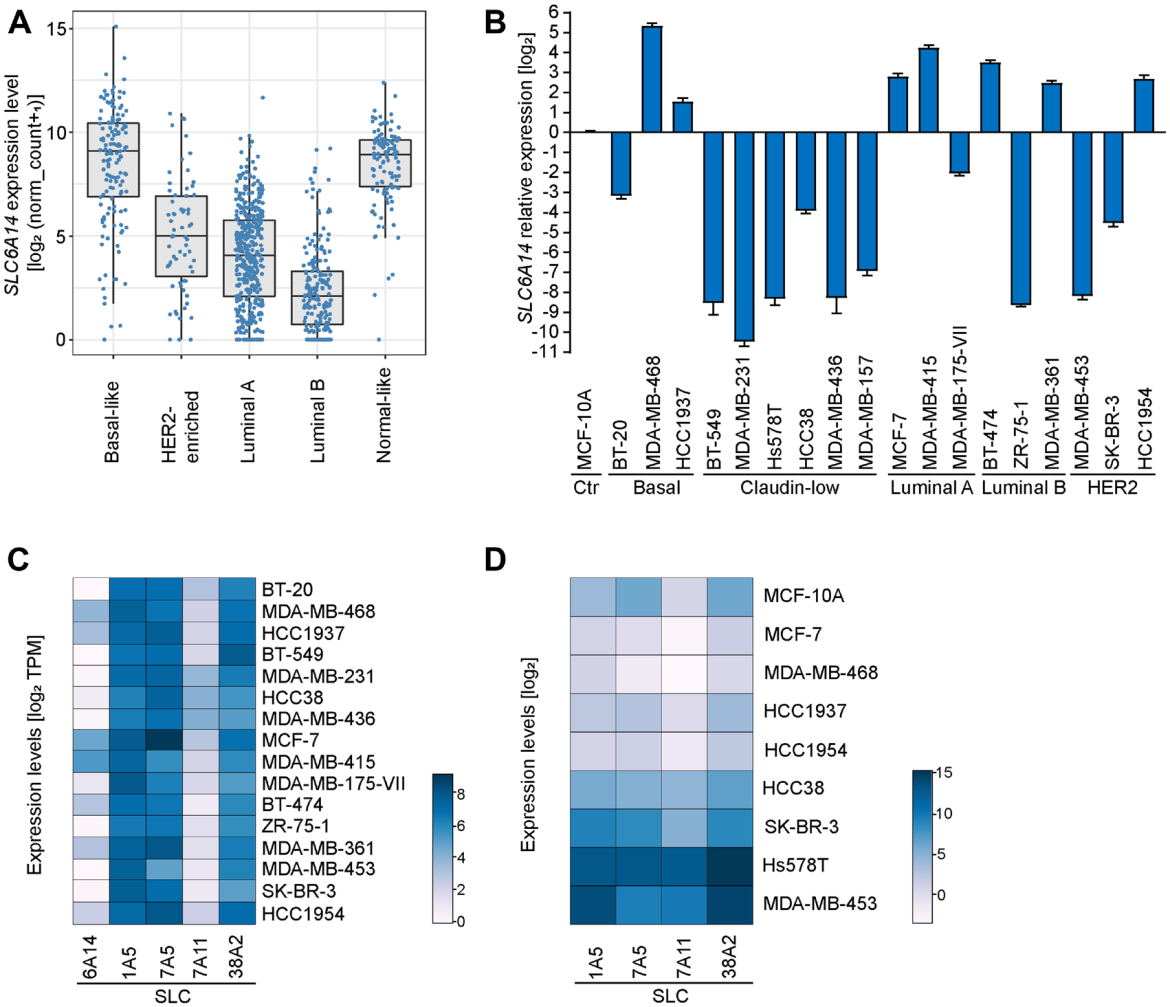
SLC6A14 expression levels in human breast tumors and cancer cell lines. (**A**) SLC6A14 mRNA levels in human breast cancer samples divided by subtypes. Data analysis performed using the Breast Invasive Carcinoma (BRCA) dataset from TCGA with available PAM50 annotations (*n* = 522). Transcript levels are pan-cancer normalized. *P*-values were calculated using a Mann–Whitney *U* test (each subtype compared to the basal-like subtype [*p* = 3.8e-11 HER2-enriched; *p* < 2e-16 Luminal A; *p* < 2e-16 Luminal B; *p* = 0.12 Normal-like]). (**B**) RT-qPCR of SLC6A14 mRNA levels in breast cancer cell lines divided by subtypes. Data are representative of 3 independent experiments. Error bars indicate standard deviation of technical triplicates. (**C**) Heatmap showing the expression levels of 5 genes encoding amino acid transporters in breast cancer cell lines. Data are obtained from the DepMap database (version 20Q2) and expressed as TPM (Transcript Per Million). (**D**) Heatmap showing the expression levels of 4 genes encoding amino acid transporters in selected breast cancer cell lines. Data are obtained by RT-qPCR analysis and are representative of 3 independent experiments.

To evaluate the expression levels of SLC6A14 in tumor samples we used breast cancer as a study model. Our initial analysis of SLC6A14 mRNA expression on human breast tumor samples available from The Cancer Genome Atlas database (TCGA) revealed a high variability in SLC6A14 transcript levels. Grouping the samples based on the major breast cancer subtypes defined by the PAM50 gene expression signature [13, 14], we observed the highest levels of SLC6A14 expression in the basal-like and in the normal-like subtypes (Figure 1A). The basal-like subtype comprises mainly, but not only, tumors that lack the expression of hormone receptors (ER-; PR-; Her2- or triple negative), which give rise to the highly aggressive triple negative breast cancer (TNBC). Our results suggest that in the TNBC subtype, SLC6A14 expression is particularly elevated.

Next, we assessed by RT-qPCR SLC6A14 transcript levels in a panel of breast cancer cell lines representative of five well-defined breast cancer subtypes [15] (Figure 1B). Using MCF-10A, a non-tumorigenic breast epithelial cell line, for data normalization, we confirmed the high variability in SLC6A14 expression in different breast cancer cell lines. We then compared these results to the expression data retrieved from the DepMap database and observed consistency with our analysis (Figure 1C). Previously, SLC6A14 expression has been reported to be correlated with the estrogen receptor (ER) status [16], however we did not find a significant correlation between the two variables in the tested breast cancer cell lines (*P*-value = 0.1274, Mann-Whitney *U* test) or in the breast tumors (Supplementary Figure 1A). Collectively, these results indicate that the ER may represent one out of multiple factors controlling SLC6A14 expression.

We next evaluated the expression levels of additional amino acid transporters in a smaller panel of breast cancer cell lines and human tumors (Figure 1D, Supplementary Figure 1B). We selected four transporters which are expressed at high levels in human breast tumors (Supplementary Figure 1A) and whose function has been found to be relevant in breast cancer [6, 17, 18]. Data from the DepMap database show high expression levels of SLC1A5, SLC7A11, SLC38A2, and SLC7A5 in the selected cancer cell lines (Figure 1C). In our analysis, we compared the expression levels of the four transporters to those of SLC6A14 in each cell line. We observed in one group of cancer cell lines (MCF-7, MDA-MB-468, HCC1937 and HCC1954) no dramatic changes in the expression of other transporters as compared to SLC6A14 levels, whereas in an another group of cell lines (HCC38, SKBR3, Hs578T, MDA-MB-453) with typically lower basal levels of SLC6A14 we detected increased expression levels of the four transporters (Figure 1D). In summary, expression data from human tumors and cancer cell lines show that SLC6A14 is overexpressed in a subset of tumors and suggest that, in these contexts, its preferential expression can be sufficient to fulfill tumor metabolic requirements.

### Genetic inhibition of SLC6A14 does not impact cell proliferation nor induces cell death under normal culture conditions

**Figure 2:**
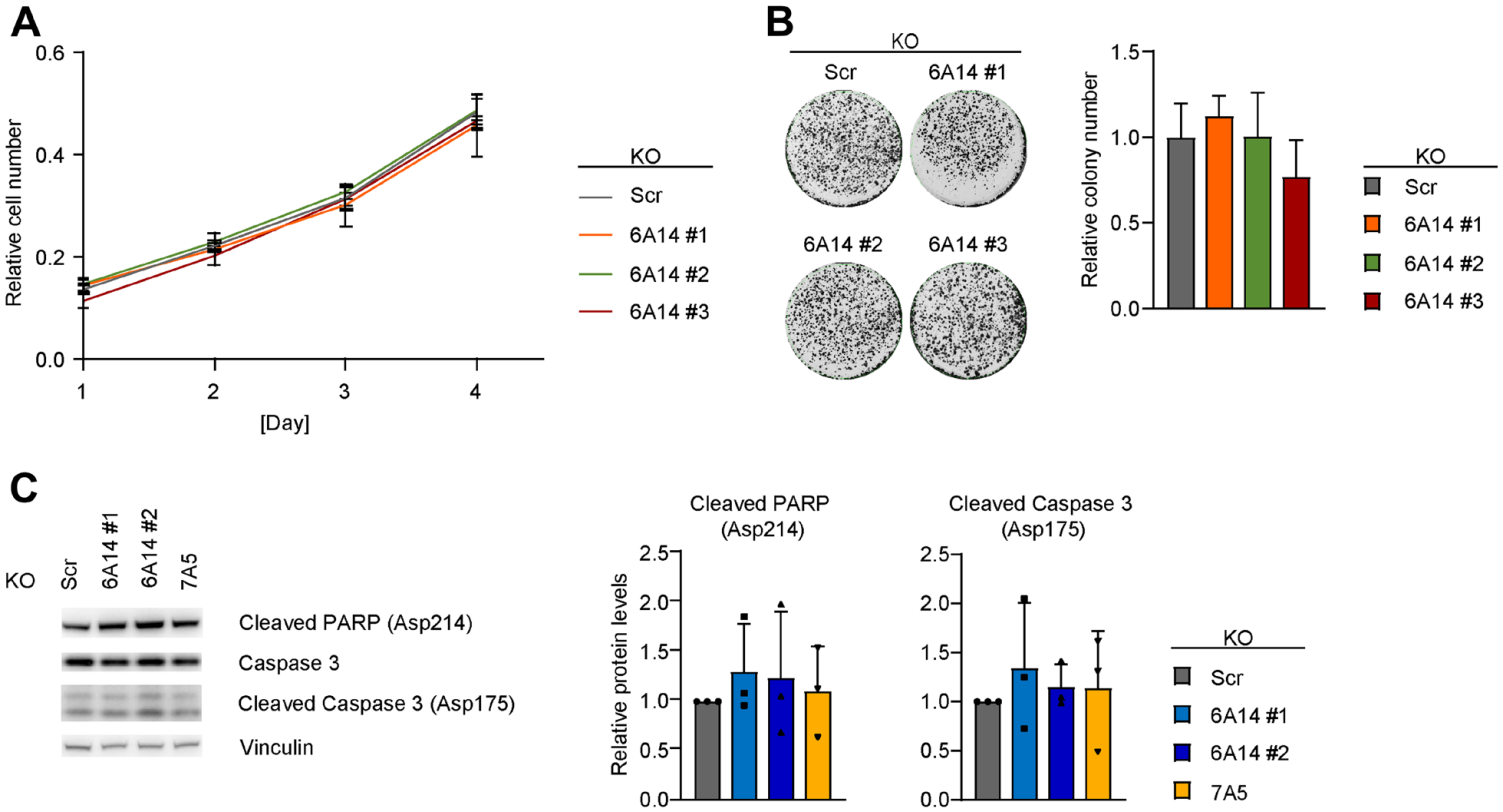
Genetic inhibition of SLC6A14 does not have a major impact on cell viability. (**A**) Cell number quantification of scrambled and SLC6A14 KO MDA-MB-468 cell lines. Equal number of cells were plated, stained with crystal violet at each indicated time point and quantified by reading the absorbance at 550 nm. Data represent mean ± standard deviation of 3 independent experiments. (**B**) (Left) Representative colony formation assay image of scrambled and SLC6A14 KO MDA-MB-468 cell lines. (Right) Colony number quantification. Data represent mean + standard deviation of 4 independent experiments. (**C**) (Left) Immunoblot showing levels of apoptotic markers in inducible MDA-MB-468 KO cell lines after 5 days Dox-induction. (Right) Immunoblot quantification. Data represent mean + standard deviation of 3 independent experiments.

To test the effects of SLC6A14 deficiency we first focused on one of the highest SLC6A14 expressing cell lines, MDA-MB-468. In this cell line, we established CRISPR/Cas9-based genetic loss of function models by generating stable knock-out (KO) clones or an inducible KO system to measure effects in a time-controlled manner by stable integration of specific guide RNAs (gRNAs) and doxycycline-mediated induction of Cas9 expression. Due to the lack of specific antibodies against SLC6A14, the KO efficacy and efficiency were tested in both systems by Sanger sequencing and by RT-qPCR, which allowed us to identify the specific CRISPR/Cas9-mediated genomic event in each clone and to confirm that decreased SLC6A14 expression levels can be used as a measure of the genetic loss of function, respectively (Supplementary Figure 2A and 2B). The efficiency of inducible KO models for SLC1A5 and SLC7A5 was tested by immunoblot (Supplementary Figure 2C). Using the stable KO clones, that mimic complete and chronic loss of function of SLC6A14, we assessed the proliferation rates of the cells. In three independent SLC6A14 KO clones we observed no marked changes in their proliferation rates or in their ability to form colonies compared to a control (non-targeting scrambled gRNA) KO clone (Figure 2A and 2B). In addition, the induction of SLC6A14 KO did not lead to any significant activation of apoptosis, as indicated by the cleavage of two apoptotic markers Poly (ADP-Ribose) Polymerase-1 (PARP-1) and Caspase-3. Similar results were observed when inducing the KO of the amino acid transporter SLC7A5 which is expressed at levels comparable to those of SLC6A14 in MDA-MB-468 cells (Figure 2C). Overall these results indicated that, although expressed at high levels, SLC6A14 function appears to be dispensable in MDA-MB-468 cells under normal culture conditions.

### SLC6A14 expression increases upon amino acid stress

**Figure 3:**
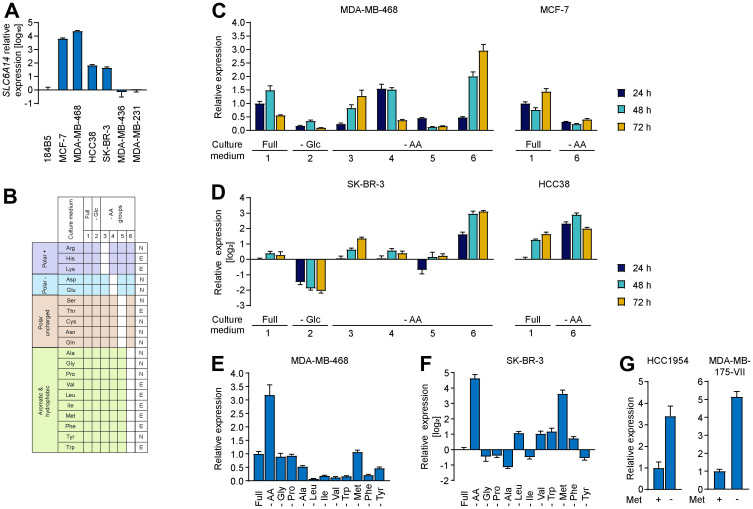
Changes in SLC6A14 expression levels upon amino acid stress. (**A**) RT-qPCR of SLC6A14 mRNA levels in selected breast cancer cell lines. Error bars indicate standard deviation of technical triplicates. (**B**) Schematic indicating the culture conditions used for characterizing the transcriptional stress response. The 20 amino acids are grouped based on their chemical properties (left column) The white boxes indicate the metabolites not included in the medium formulation. In the right column amino acids are classified as essential, E, or non-essential, N. (**C** and **D**) RT-qPCR of SLC6A14 mRNA levels in the indicated breast cancer cell lines upon metabolic stress. Data are representative of 3 independent experiments. Error bars indicate standard deviation of technical triplicates (*n* = 3 (MDA-MB-468; SK-BR-3; HCC38), *n* = 2 (MCF-7)). (**E** and **F**) RT-qPCR of SLC6A14 mRNA levels in MDA-MB-468 and SK-BR-3 breast cancer cell lines upon removal of the indicated amino acids for 72 h. Data are representative of 3 independent experiments. Error bars indicate standard deviation of technical triplicates. (**G**) RT-qPCR of SLC6A14 mRNA levels in HCC1954 and MDA-MB-175-VII breast cancer cell lines in the presence or absence of methionine for 72 h. Error bars indicate standard deviation of technical triplicates.

We reasoned that the lack of major impact on cell proliferation or cell death in response to SLC6A14 KO may be due to the rich culture conditions under which the experiments were performed that could potentially mask specific responses that occur only under stress conditions. Transcriptional regulation of genes encoding amino acid transporters is a common mechanism occurring in response to metabolic stressors [19] and inspection of SLC6A14 promoter [20] revealed the presence of ATF4 binding sites, indicators of consensus amino acid response elements (AARE). We therefore hypothesized that SLC6A14 expression might be specifically elevated upon stress as a compensatory mechanism to allow cells to increase their nutrient uptake. Hence, inhibiting the transporter under these conditions may represent a beneficial strategy to trigger metabolic imbalance. In order to test this hypothesis and assess changes in SLC6A14 mRNA levels, we selected three groups of cell lines with varying levels of basal SLC6A14 expression: i) first group is composed of cell lines which show high SLC6A14 expression levels (MDA-MB-468, MCF-7) ii) the second group expresses low levels of SLC6A14 (HCC38, SK-BR-3) iii) and the third group consists of two cell lines (MDA-MB-436, MDA-MB-231) that do not express the transporter (Figure 3A). In order to trigger evident transcriptional responses, we performed complete starvation of different metabolites. Specifically, we tested six distinct stress conditions by culturing those cell lines in media based on the RPMI formulation and lacking either glucose or four different groups of amino acids, according to their side chain chemical properties (polar positive, polar negative, polar uncharged, hydrophobic and aromatic) (Figure 3B). Each of these stress conditions were in addition coupled to hypoxia and measured in a time-dependent manner after 24, 48 and 72 hours. We confirmed the induction of hypoxia by increased expression of Hypoxia Inducible Factor (HIF)-1α target genes (Supplementary Figure 3A), however under these conditions we did not detect any increase in SLC6A14 expression (Supplementary Figure 3C and 3D). The gene expression of markers of cell cycle arrest such as CDKN1A (encoding p21) and CDKN1B (encoding p27) was induced by hypoxia and starvation separately, however their levels were attenuated in response to combined amino acid and hypoxic stress, possibly due to a transcriptional shut-down (Supplementary Figure 3B). We observed global changes in the pattern of SLC6A14 expression under metabolic stress conditions in all the SLC6A14-positive cell lines (Figure 3C and 3D), whereas no changes were detected in the SLC6A14-negative ones (data not shown). In general, glucose starvation induced a transcriptional downregulation of SLC6A14. A similar effect was observed in the medium formulation 5, which lacks the polar uncharged amino acids. SLC6A14 levels were found to be unaltered when the cells were cultured either in the presence of the complete medium (condition 1) or in the medium lacking the negatively charged amino acids (aspartate and glutamate). These results are in agreement with the fact that aspartate and glutamate uptake is dispensable, at least in the presence of other amino acids [21]. Interestingly, we found in all the tested cell lines, except MCF-7, a consistent increase in SLC6A14 expression in the culture condition that lacks the hydrophobic and aromatic amino acids (depicted as condition 6). Although the trends of transcriptional changes upon starvation of different amino acid groups were overall similar in all the cell lines, the magnitude of changes was much more pronounced in the SK-BR-3 and HCC38 cell lines that express lower levels of SLC6A14 under basal culture conditions. These results highlight therefore how the expression profile of SLC6A14 undergo dynamic changes in response to metabolic perturbations.

In order to obtain a more comprehensive overview, we also evaluated changes in the expression levels of three additional transporter-coding genes (SLC1A5; SLC7A5 and SLC38A2), focusing on MDA-MB-468 and SK-BR-3 cell lines, in which we observed the most fluctuations in SLC6A14 expression levels (Supplementary Figure 3C and 3D). Analysis of the expression levels of the three transporters confirmed the dynamic transcriptional responses to stress with major changes found in the absence of each amino acid group except glutamate and aspartate and a blunted transcriptional response under hypoxic conditions. In addition, we combined SLC6A14 KO and starvation of the hydrophobic and aromatic amino acids and measured the gene expression levels of these transporters to evaluate a potential compensatory effect. We did not observe a major increase in the expression levels of SLC1A5 and SLC38A2, however, a minor increase in the levels of SLC7A5 was visible (Supplementary Figure 4A). In conclusion, we found that the lack of hydrophobic and aromatic amino acids (condition 6) triggers transcriptional activation especially for SLC6A14 expression, while the expression pattern of other amino acid transporter appears to be more heterogeneous in response to stress.

### Methionine starvation induces SLC6A14 mRNA upregulation

To narrow down the candidate metabolic stressor, we evaluated how the starvation of each single amino acid in the hydrophobic and aromatic amino acid group impacts on the expression levels of SLC6A14. We collected cells after 72 hours of starvation since we observed the highest transcriptional changes at this time point. We found that, in the MDA-MB-468 cell line, which expresses high basal levels of SLC6A14, none of the single amino acid starvation was able to recapitulate the upregulation observed upon combined starvation, suggesting that the absence of multiple amino acids can be responsible for an increase in gene expression. While single starvation of seven amino acids (Ala, Leu, Ile, Val, Trp, Phe, and Tyr) led to SLC6A14 transcript downregulation, we could identify three conditions in which SLC6A14 mRNA levels are stably maintained (Gly, Pro, and Met starvation) and that are likely to contribute to the upregulation achieved by the combined starvation (Figure 3E). As a comparison we also followed SLC7A5 expression levels in response to the single amino acid starvation and observed that the highest levels of increase were achieved by methionine starvation alone (Supplementary Figure 3E). We then analyzed SK-BR-3 cell line under these conditions since we found a higher degree of the transcriptional changes upon stress in cell lines that express low basal levels of SLC6A14. Consistent with this observation, we were able to detect more pronounced effects in this cell line also upon starvation of single amino acids (Figure 3F). Although the two cell lines showed some differences in terms of their transcriptional response, e.g., SLC6A14 upregulation in SK-BR-3 upon Leu, Val and Trp starvation, rather than downregulation as observed in MDA-MB-468, in both cases SLC6A14 transcript levels were responsive to methionine starvation. Strikingly, we found that in SK-BR-3 cells methionine starvation alone leads to a strong upregulation of SLC6A14 whereas, similar to what we observed in MDA-MB-468, the mRNA levels of another amino acid transporter gene (SLC7A5) show a rather unspecific increase upon several metabolic insults (Supplementary Figure 3F). We confirmed the induction of SLC6A14 expression upon methionine starvation in two additional cell lines: HCC1954 and MDA-MB-175-VII (Figure 3G). These results indicate that methionine starvation can be used as a metabolic stress condition to evaluate the relevance of amino acid transporter function and the impact of their inhibition for two reasons: on the one hand, it leads to a specific upregulation or stabilization of SLC6A14 mRNA levels, on the other hand, it triggers the simultaneous upregulation of additional transporters, as exemplified by SLC7A5.

### AMPK is activated in the absence of SLC6A14 by methionine starvation

**Figure 4:**
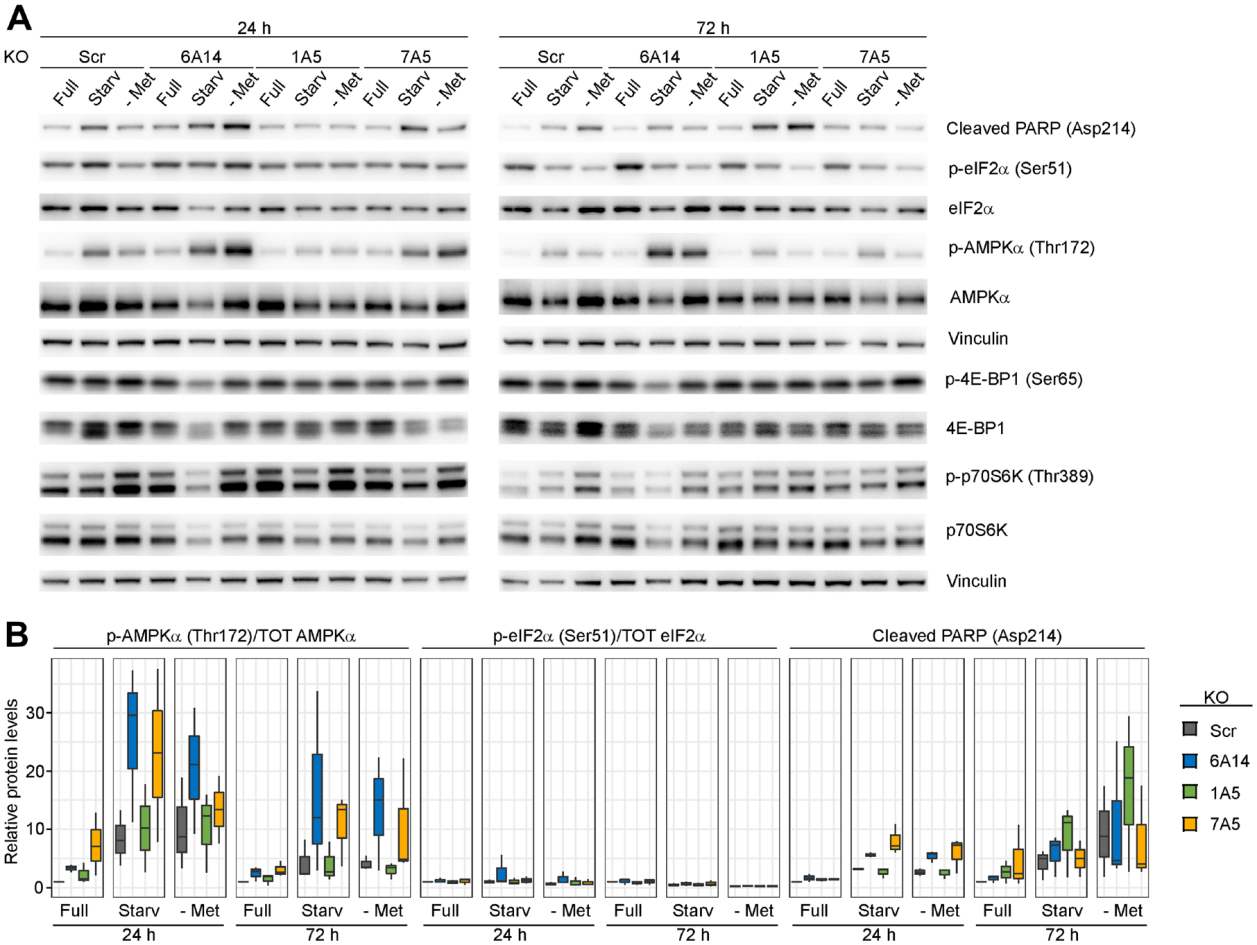
Evaluation of stress response markers in the absence of amino acid transporters. (**A**) Immunoblots of inducible MDA-MB-468 KO cell lines upon metabolic stress. Cells were induced with doxycycline for 4 days, plated and cultured for 24 h or 72 h in the indicated media: Full = complete medium; Starv = medium without aromatic and hydrophobic amino acids; - Met = medium without methionine. (**B**) Immunoblot quantification. Boxplots represent values from three independent experiments. In the boxplots, centerlines mark the medians, box limits indicate the 25th and 75th percentiles, and whiskers extend to 5th and 95th percentiles.

To test the effects of SLC6A14 inhibition when cells are subjected to amino acid stress, we assayed the activation of stress response markers in order to identify potential differential responses. We considered for our analysis three major hubs on which stress responses impinge: i) the apoptotic pathway, using the cleavage of PARP-1 at position 214 as a read-out of caspase-3 activity; ii) the integrated stress response, defined by the activating phosphorylation of eukaryotic translation initiation factor 2A (eIF2α) on which multiple stress stimuli converge by activation of upstream kinases and which, in turn, results in the inhibition of the 5′ Cap-dependent protein synthesis as well as the activation of the transcription factor ATF4 [22]; iii) Activation of AMPK, as determined by the phosphorylation levels of its catalytic α subunit. Energetic stress resulting in changes in the intracellular AMP to ATP ratio represents the major stimulus activating AMPK, which, in turn, controls the function of a plethora of targets in order to maintain energetic homeostasis [23]. We tested how inducible MDA-MB-468 KO cell lines respond to metabolic stress upon genetic inhibition of three amino acid transporters (SLC6A14; SLC1A5; SLC7A5) (Figure 4A and 4B). As expected, we detected over time in the control KO cell line increased cleavage of PARP-1, indicator of apoptosis induction, following both combined amino acid starvation (condition 6) or methionine starvation alone. However, we found that inhibition of SLC6A14 leads to a higher induction of apoptosis compared to the control already after 24 h of starvation without further increase at later time points. The same effect was observed upon SLC7A5 KO, while a delayed response occurred upon SLC1A5 KO. In all cases, inhibition of amino acid transporters exacerbates the apoptotic response due to metabolic stress, indicating that cells become dependent on the amino acid transporter function under these conditions.

We next assessed how levels of phosphorylated eIF2α change under the same conditions. Amino acid deprivation is known to induce activation of the integrated stress response regulator general control non-derepressible 2 (GCN2), which is activated by uncharged tRNAs. GCN2, in turn, phosphorylates eIF2α. Surprisingly, we did not observe any significant change of phosphorylated eIF2α levels in any of the tested conditions. A time course experiment performed culturing MDA-MB-468 cells in the absence of methionine showed only a mild increase of phospho-eIF2α after short-term starvation (Supplementary Figure 4B). We predict that high basal levels of activated eIF2α observed in this cell line, in agreement with a previous report [24], may prevent its further phosphorylation and activation.

Finally, we analyzed changes in the AMPK activity upon metabolic stress, assessed by the phosphorylation on Thr172 of its α subunit. We detected an increased activation of AMPK in the control cell line, both after 24 and 72 h of single methionine starvation or starvation of the whole group of hydrophobic and aromatic amino acids. Interestingly, in the SLC6A14 KO cell line, we observed a further increase in the levels of phosphorylated AMPKα which was enhanced upon starvation. In order to account for the experimental variability, we confirmed these results also in three independent stable SLC6A14 KO cell lines (Supplementary Figure 4C). A mild increase was also evident in SLC7A5 KO cells, whereas no obvious differences to the control cell line were observed upon genetic inhibition of SLC1A5.

Since AMPK activation is known to inhibit the activity of the mammalian target of rapamycin (mTOR) pathway, we additionally measured phosphorylated levels of the two mTOR targets p70S6 kinase (p70S6K) and 4E-binding protein 1 (4E-BP1). Surprisingly, we observed in all cell lines decreased phosphorylation of both proteins only upon combined amino acid starvation but not when cells were starved for methionine alone (Figure 4A). Thus, in these cells, the mTOR activation status does not directly correlate with AMPK activation. To gain more insights about AMPK activity in the MDA-MB-468 cell line, we evaluated changes in its phosphorylated levels when the cells were cultured under hypoxic conditions, a known AMPK activating stimulus [25]. However, we found that, in this cell line, the metabolic stress induced by starvation of a group of 10 amino acids (condition 6) represents the major contributor to AMPK activation, which is not further increased upon hypoxia (Supplementary Figure 4D).

Overall, these results indicate that metabolic stress conditions, that lead to increased expression of SLC6A14, induce an intracellular response which mainly impinges on AMPK activation. In addition, coupling amino transporter inhibition to metabolic stress enhances this effect.

### AMPK activation is a metabolic liability in SLC6A14-deficient cells

Since we found that methionine starvation alone represents a sufficient stimulus to induce AMPK activation and that such response increases by blocking SLC6A14, we evaluated if changes in the intracellular energy status under these conditions may account for the heightened AMPK activity. We performed therefore steady-state measurements of intracellular levels of the adenine nucleotides AMP, ADP, and ATP after induction of SLC6A14 KO in cells cultured for 24 h in the presence or in the absence of methionine. Interestingly, we observed a mild, though nonsignificant, increase in the ADP/ATP ratio upon methionine starvation (Supplementary Figure 5A). Additionally, we tested changes in the intracellular amino acid levels (except for tryptophan) under the same conditions (Supplementary Figure 5B). We detected minor compensatory effects due to increased levels of few amino acids upon methionine starvation, in a SLC6A14-independent way, while a strong reduction in the intracellular methionine levels was observed upon starvation, confirming the efficacy of the treatment. Finally, we evaluated changes in the levels of the methionine-derived metabolites S-adenosylmethionine (SAM) and S-adenosylhomocysteine (SAH). A mild reduction in the levels of both metabolites was observed upon methionine starvation, however the SAM/SAH ratio, indicator of the intracellular methylation status, was not significantly changed (Supplementary Figure 5C). Therefore, under these experimental conditions, steady-state measurement of intracellular metabolite concentrations did not allow us to pinpoint a specific stimulus which could mediate AMPK activation. However, due to the known function of AMPK as a metabolic rheostat, it is possible that its activation allows cells to maintain a metabolic balance and prevents them from undergoing apoptosis.

**Figure 5:**
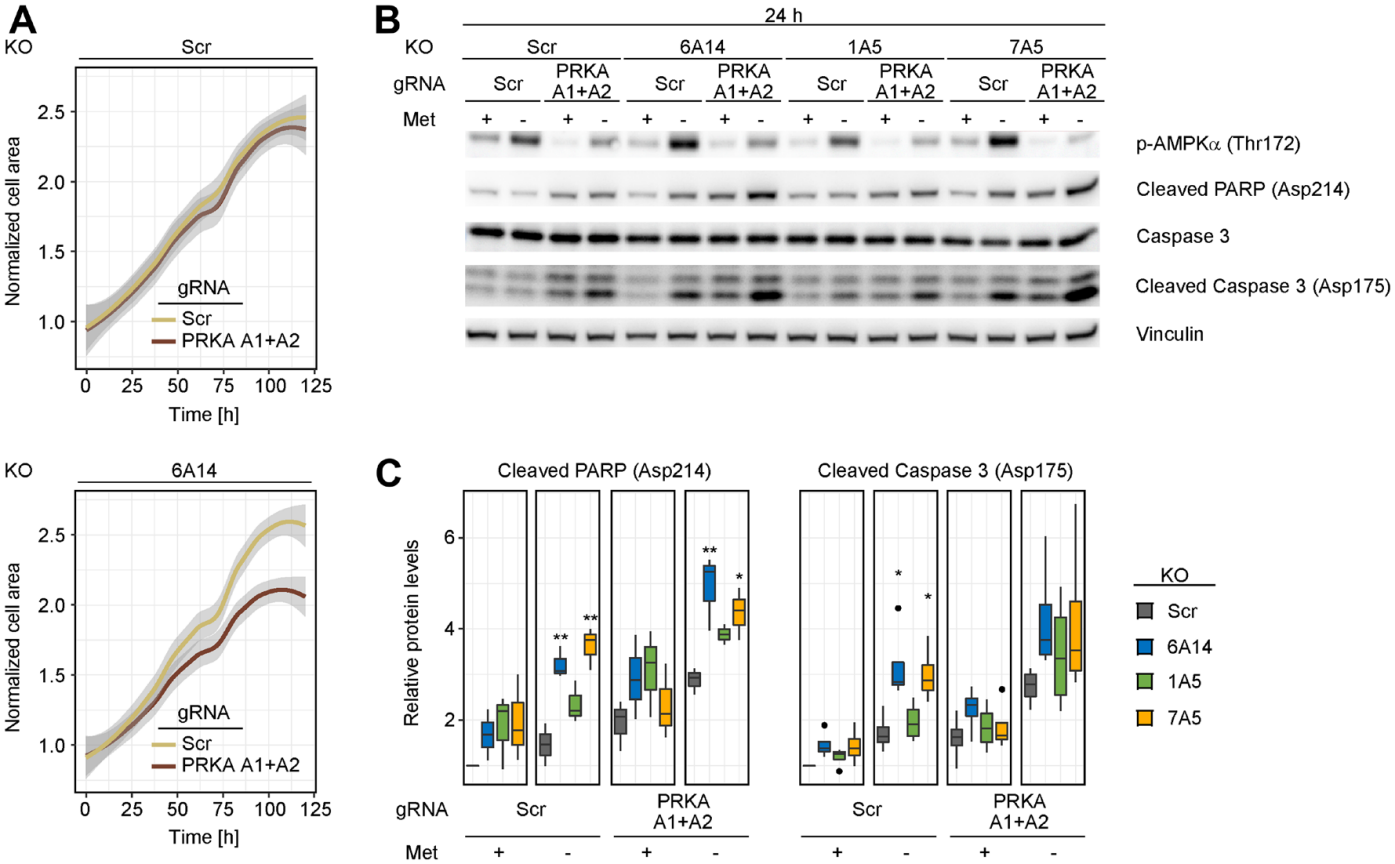
Combined depletion of AMPK and SLC6A14 induces cell death in response to methionine starvation. (**A**) Cell proliferation analysis of inducible MDA-MB-468 KO cell lines upon AMPK inhibition. Control and SLC6A14 inducible KO cells were treated with doxycycline for 4 days and transfected with a scrambled gRNA or gRNAs targeting PRKAA1 and PRKAA2. Three days post transfection equal number of cells were plated on a 96-well plate in full medium and the cell proliferation was followed over 5 days using the Incucyte Imaging system. The images were analyzed using the ImageJ software by calculating the area occupied by the cells. Data represent mean of 3 replicates. The grey shade area indicates the 95% confidence interval. Statistical significance was tested with a Mann–Whitney *U* test. Significant *P*-values were obtained in the SLC6A14 KO cell line (*p* ≤ 0.01 at 36 h, 48 h, 60 h, 72 h, 84 h, 96 h, 120 h; *p* ≤ 0.001 at 108 h). (**B**) Immunoblots of inducible MDA-MB-468 KO cell lines upon metabolic stress and AMPK inhibition. Inducible KO cell lines were treated as in (A). Three days post transfection equal number of cells were plated on a 6-well plate and cultured for 24 h in the indicated media: Full = complete medium; - Met = medium without methionine. (**C**) Immunoblot quantification. Boxplots represent values from ≥ 3 independent experiments. In the boxplots, centerlines mark the medians, box limits indicate the 25th and 75th percentiles, and whiskers extend to 5th and 95th percentiles. *P*-values are calculated based on an ANOVA test followed by *Tukey* pairwise comparisons (^**^ *p* ≤ 0.01; ^*^ *p* ≤ 0.05).

Increased levels of phosphorylated AMPK in SLC6A14 KO cells challenged with amino acid stress prompted us to test whether blocking AMPK activation can be a vulnerability under these conditions. To avoid confounding unspecific effects that are reported for AMPK inhibitors [26], we used a genetic deletion background to test our hypothesis. Initially, we analyzed how cells respond to AMPK inhibition when cultured under metabolically unperturbed conditions by measuring changes in their proliferation rate over time. For this experiment, we transfected gRNAs targeting PRKAA1 and PRKAA2, which encode AMPK α1 and AMPK α2 subunits, respectively, in our isogenic cell lines. Consistent with the fact that SLC6A14 KO alone induces a mild activation of AMPK, we observed a moderate decrease in the cell proliferation rate only in the SLC6A14 KO cell line. This effect became more pronounced after 3 days, possibly due to metabolite consumption in the culture medium which triggers metabolic stress (Figure 5A). We next followed the cleavage of PARP-1 and caspase-3 as markers of apoptosis. Consistent with our previous observations, KO of SLC6A14 as well as SLC7A5 in combination with methionine starvation led only to a slight increase in the cleaved PARP-1 and caspase-3 levels. However, when we combined AMPK depletion with transporter KO (i.e., triple KO cell line) in the presence of methionine starvation, we observed heightened levels of the cleavage products, indicating an increased rate of apoptosis (Figure 5B and 5C). This combination seems to be most pronounced upon SLC6A14 KO, in line with our previous results demonstrating the highest increase of AMPK activation in SLC6A14 KO cells. Altogether, our data indicate that AMPK activation is a metabolic vulnerability in SLC6A14-deficient cells that can be exploited as a therapeutic approach to drive unbalanced metabolism in starved tumor cells.

## DISCUSSION

Targeting tumor metabolism has been explored as a strategy to inhibit tumor growth and proliferation due to the elevated metabolic requirements of cancer cells compared to their normal counterparts [1]. In such context, blocking the uptake of amino acid by inhibiting their transporters may represent a viable approach to starve tumors from their widely used metabolites [27]. The effects of amino acid transporter inhibition on reducing tumor viability have been shown in a subset of cancers demonstrating the potential efficacy of such approaches [8, 12, 28]. For instance, the SLC7A5 (LAT1) inhibitor JPH203 has been tested in clinical trials showing promising effects for the treatment of solid tumors refractory to standard therapy [29]. Owing to its unique properties, such as the broad substrate specificity, the amino acid transporter SLC6A14 represents an attractive therapeutic target. However, targeting amino acid transporters as a single therapeutic option has been shown to be inefficient as a result of tumor plasticity and the intrinsic redundancy of the transporter system [30, 31]. Therefore, drug combination regimens are required to successfully block both nutrient acquisition pathways and potential metabolic adaptations [9, 27]. An important step towards this goal has been initiated by the RESOLUTE consortium, which aims to de-orphanise members of the solute carrier (SLC) family by generating tools and data at large scale [32]. Building on this, understanding the right targeting conditions will be critical for developing effective therapies.

In this study, we extend the knowledge about SLC6A14 function by exploiting for the first time the combination of metabolic stress coupled to the transporter inhibition and elucidate the molecular responses elicited in breast cancer cells. By combining human breast cancer data analysis and a broad panel of breast cancer cell lines, we show that SLC6A14 is expressed at highly heterogenous levels. In contrast to previous observations [16], we found that SLC6A14 can be expressed in ER negative tumors. In particular, exploring the TCGA data, we show that SLC6A14 expression is highest in the basal-like subtype, which typically gives rise to the triple negative breast cancers (TNBC), the most aggressive type of breast tumors with no effective treatment strategies. These data suggest that SLC6A14 can be considered as a potential therapeutic target also in this tumor subtype. The genetic deletion of SLC6A14 in MDA-MB-468 cells, which express the highest SLC6A14 levels compared to the other tested cell lines, did not lead to any gross phenotypic effect, nor detectable changes in the intracellular amino acid content, indicating that culture conditions that mimic a nutrient-rich environment can possibly mask the transporter inhibitory effect since the cells would still be able to uptake the necessary metabolites by using other transporters.

Metabolic stress, such as amino acid starvation, triggers intracellular adaptive responses which allow tumor cells to survive under harmful conditions. Transporter gene upregulation represents a common response to starvation in order to optimize the uptake of limited nutrients [33]. In fact, our transcriptional analysis performed under metabolic stress conditions highlights the complexity of the transporter expression regulation. We report here cell- and condition-specific patterns of expression relative to three other relevant transporters (SLC1A5, SLC7A5, SLC38A2), providing a ground for future evaluation of the function of these transporters under stress. Interestingly, we found that SLC6A14 expression was mostly affected by the starvation of hydrophobic and aromatic amino acids, for which the transporter has the highest affinity [10]. This group comprises 10 different amino acids, among which starvation of methionine showed the highest impact on SLC6A14 expression.

Methionine is an essential amino acid that is catabolized and recycled in the methionine cycle. It is the only source of the universal methyl donor S-adenosyl-methionine (SAM), that is essential for methylation reactions in the cell, modulating gene expression through methylation of DNA, RNA and histones. Beyond this function, methionine also contributes to essential metabolic pathways including polyamine synthesis, maintenance of the redox status and control of protein synthesis [34]. Interestingly, while untransformed tissues are able to sustain exogenous methionine depletion, tumor cells can become dependent on methionine availability [35]. Taking advantage of this therapeutic window, several approaches targeting methionine metabolism are under investigation, for instance by altering the dietary methionine intake [34, 35]. Dietary methionine restriction has been also suggested to increase sensitivity to certain cytotoxic agents, such as cisplatin and doxorubicin [36]. Notably, in TNBC models, methionine restriction is shown to increase sensitivity of chemotherapeutic agents and prevent metastases [36, 37]. Given that SLC6A14 is highly expressed in TNBCs, these tumors might be mostly affected by methionine deprivation combined with SLC6A14 depletion. Indeed MDA-MB-468, a cell line derived from TNBC, is reported to be methionine sensitive [38]. Taken together, we propose that, in this context, SLC6A14 inhibition may represent a metabolic vulnerability.

**Figure 6:**
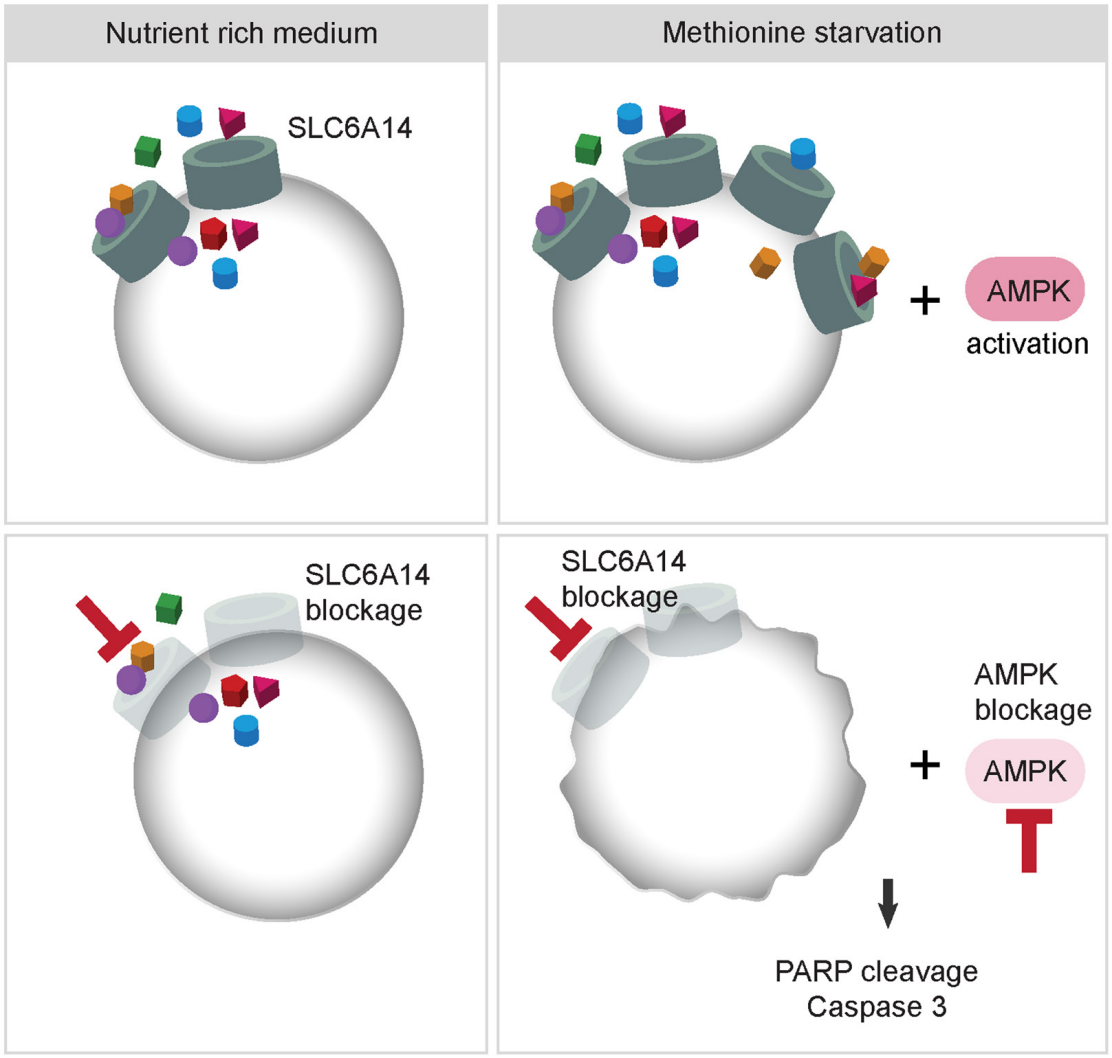
Model describing the effects of combined inhibition of SLC6A14 and AMPK. Under nutrient-rich conditions SLC6A14 is expressed and is available to transport 18 different amino acids. Under metabolic stress, such as methionine starvation, SLC6A14 levels are increased allowing the cells to cope with the stress conditions. In addition, as a compensatory mechanism AMPK is activated. Knock-out of SLC6A14 in the absence of additional metabolic stress is tolerated, however, combined knock-out of SLC6A14 and AMPK together with methionine starvation leads to increased apoptosis.

Finally, we observed a marked activation of the energy stress sensor AMPK upon combined methionine deprivation and SLC6A14 inhibition, consistent with the idea that cells activate compensatory mechanisms to survive metabolic stress. AMPK’s role in tumorigenesis is context-dependent and both pro- and anti-tumorigenic properties have been linked to its function [39]. The AMPK-mediated inhibition of anabolic processes may provide tumor cells the plasticity necessary to survive metabolic stress, which is often occurring in rapidly growing tumors. Thus, under certain conditions, AMPK exerts a tumor promoting function, which has been demonstrated also in the context of breast cancer [40, 41]. Supporting these notions, we show here that under methionine starvation coupled to SLC6A14 deletion, AMPK activation supports cell viability, thus, its genetic inhibition upon stress induces tumor cell death (Figure 6). Under the tested experimental conditions, we did not observe major changes in the intracellular energy status nor in the amino acid levels, which could account for AMPK activation. However, we note that the steady-state measurements performed in this study may not allow us to detect dynamic metabolic changes which can be triggered by methionine starvation. Flux analyses may in this case help to elucidate the fate of intracellular methionine upon starvation. We indeed observed, on the one side, a strong reduction in the intracellular methionine content 24 h after its removal from the culture medium. On the other side, we did not detect any changes in the levels of two tested methionine-derived metabolites (SAM and SAH), possibly indicating that the already available pool of intracellular methionine is used to support methylation reactions. Another possibility is that AMPK activation is induced in a nucleotide-independent way, e.g., due to increased ROS levels [42]. Finally, we noticed that methionine starvation in MDA-MB-468 cells did not provide a stimulus sufficient to inhibit mTORC1 activity, even though in the presence of activated AMPK. Is it possible that the presence of the remaining amino acid in the culture medium, some of which are known mTORC1 activators [43], can counterbalance the AMPK-mediated inhibitory effects. In summary, although a more comprehensive understanding of the molecular events driven by AMPK activation is required, our results help to predict a suitable framework of conditions in which AMPK inhibition can be synthetically lethal, i.e., SLC6A14 inhibition combined with methionine deprivation, and support therefore further efforts in developing specific AMPK inhibitors. However, we are aware that our model requires a deeper evaluation in an *in vivo* setting. Since we observed AMPK activation occurring also upon inhibition of SLC7A5, which is in turn upregulated by methionine starvation, it is plausible that this mechanism may not be limited to SLC6A14 and warrants further investigation as a cancer vulnerability upon inhibition of nutrient acquisition pathways. On the other hand, we cannot exclude that other stress combinations would impinge on the same regulatory axis and trigger a similar apoptotic response upon AMPK inhibition. A deeper evaluation of additional stress combinations will therefore help us to shed light on this complex regulation.

## MATERIALS AND METHODS

### Cell lines and cell culture

All cell lines were obtained from ATCC and cultured according to ATCC recommendations. All culture media were obtained from Gibco and supplemented with 10% Fetal Bovine Serum (Gibco, 10270) and 1% antibiotic/antimycotic solution (Biotrend Chemikalien GmbH, AAS-B). Starvation experiments were performed by culturing cells in media with the same composition of RPMI 1640 (except that for the addition of alanine). The complete medium composition is reported in the Supplementary Table 1. FBS used for the starvation experiments was dialyzed using a 3,5 kD molecular weight cut off membrane (SERVA Electrophoresis, 44201.01). Hypoxia was induced using a hypoxia incubator chamber (StemCell Technologies) filled with the following gas mixture: 1% O_2_; 5% CO_2_; 94% N_2_.

### Colony formation assay

Cells were plated in equal numbers in six-well plates (5000 cells per well). After 15 days colonies were stained with 0,2% crystal violet and the colony number was quantified using the ImageJ software.

### Cell proliferation analysis

Cell proliferation was monitored over time using the Incucyte S3 scanner (Essen Biosciences). Phase contrast images were acquired every 12 h using a 10× objective. Individual images were analyzed using the ImageJ software with an in house written macro. Briefly, the area occupied by cells is calculated using image segmentation based on the brightfield images.

### RNA isolation and RT-qPCR

RNA was isolated using RNeasy Plus Mini kit (Qiagen), according to manufacturer’s instructions. cDNA synthesis was performed using the qScript cDNA Synthesis Kit (VWR International). qPCR was carried out using QuantStudio 3 Real-Time PCR System, following manufacturer’s instructions. The oligonucleotide list is provided in the Supplementary Table 2. 36B4 was used as housekeeping gene for internal normalization.

### Immunoblot

Cells were lysed in RIPA buffer (Cell Signaling, 9806S) supplemented with 0,2% SDS, PMSF and protease/phosphatase inhibitor cocktail (Cell Signaling, 9806S). Twenty μg of whole cell lysates were resolved on NuPAGE 4–12% Bis-Tris Protein Gels (Invitrogen) and transferred onto PVDF membranes (neolab Migge GmbH, IPFL00010). Membranes were blocked in 5% BSA and incubated overnight with primary antibodies (Supplementary Table 3). Anti-mouse or anti-Rabbit IgG, HRP-linked Antibody (Cell signaling, 7076; 7074) were used as secondary antibodies and signals were detected using the Vilber FUSION FX7 imaging system (Vilber Lourmat). Vinculin and β-actin served as loading controls. Band intensities were quantified using the ImageJ software.

### Knock-out cell line generation

For stable KO cell line generation, cells were infected with Edit-R Inducible Lentiviral hEF1a-Blast-Cas9 (GE Dharmacon, VCAS11227) and selected with 10 μg/ml blasticidin (Invivogen, ant-bl-05). To induce Cas9 expression, cells were treated with 1 μg/ml doxycycline (AppliChem GmbH, A2951.0025) and 24 h later transfected with pLenti-Guide-Puro vectors (Origene, GE100032) containing a non-targeting control gRNA (Scrambled) or 2 different gRNAs targeting SLC6A14, using Lipofectamine 3000 Transfection Reagent (Thermo Fisher Scientific, L3000001), in the presence of doxycycline. Cells were selected with 1 μg/ml puromycin (Invivogen, ant-pr-1) and plated for single cell clonal isolation on 96-well plates. Knock-out clones were screened by RT-qPCR and selected clones were tested by Sanger sequencing to identify the specific genomic rearrangement in each clone.

For inducible KO cell line generation, single clones were selected from cells infected with Edit-R Inducible Lentiviral hEF1a-Blast-Cas9, to obtain cells expressing high and homogeneous levels of Cas9. Selected clones were infected with pLenti-Guide-Puro vectors containing a non-targeting control gRNA (Scrambled) or gRNAs targeting SLC6A14, SLC1A5 and SLC7A5 and selected with 1 μg/ml puromycin. The knock-out was induced by treating the cells with 1 μg/ml doxycycline every 48 h. Cells were tested by RT-qPCR or immunoblot 4 days post induction. A list of the gRNAs cloned in the pLenti-Guide vector is presented in the Supplementary Table 4A.

For AMPK KO generation, inducible KO cells (Scrambled KO, SLC6A14 KO, SLC1A5 KO, SLC7A5 KO) were treated with doxycycline for 4 days to allow the KO to occur. Equal number of cells were plated on 6-well plates and transfected either with a scrambled gRNA or an equimolar mix of gRNAs targeting PRKAA1 and PRKAA2. Transfection was performed using Lipofectamine RNAiMAX Transfection Reagent (Thermo Fisher Scientific, 13778-100) and 3 μM total gRNA (1,5 μM each for PRKAA1 and PRKAA2), in the presence of doxycycline. Transfected cells were expanded after 24 h and plated for downstream assays. The original sequences of the gRNA used for transfection are listed in the Supplementary Table 4B. Single gRNA were synthetized from IDT as Alt-R CRISPR-Cas9 crRNA and annealed to Alt-R CRISPR-Cas9 tracrRNA to obtain 3 μM gRNA duplex.

### Metabolite measurements

Free amino acids were extracted from 1 × 10^6^ cells with 0.2 ml of 0.1 M HCl in an ultrasonic ice-bath for 10 min. The resulting extracts were centrifuged for 10 min at 4°C and 16.400 g to remove cell debris. Amino acids were derivatized with AccQ-Tag reagent (Waters) and determined as described in [44]. For determination of nucleotide concentrations, the extracts were derivatized with chloroacetaldehyde as described in [45] and separated by reversed phase chromatography on an Acquity HSS T3 column (100 mm × 2.1 mm, 1.7 μm, Waters) connected to an Acquity H-class UPLC system. Prior separation, the column was heated to 43°C and equilibrated with 5 column volumes of buffer A (5.7 mM TBAS, 30.5 mM KH_2_PO_4_ pH 5.8) at a flow rate of 0.6 ml min-1. Separation of adenosine derivates was achieved by increasing the concentration of buffer B (2/3 acetonitrile in 1/3 buffer A) in buffer A as follows: 1 min 1% B, 2 min 8% B, 3.2 min 14% B, 9.5 min 50% B, and return to 1% B in 1.5 min. The separated derivates were detected by fluorescence (Acquity FLR detector, Waters, excitation: 280 nm, emission: 410 nm) and quantified using ultrapure standards (Sigma). Data acquisition and processing was performed with the Empower3 software suite (Waters).

### Statistical analyses

For analysis of the TCGA data, breast adenocarcinoma dataset was downloaded from cBioPortal using CGDS R package and processed with in house developed R scripts. Statistical analysis was performed using Prism software (GraphPad) and R. The applied statistical tests are specified in the corresponding figure legends.

## SUPPLEMENTARY MATERIALS


