# Supplementary material for: Exploiting the metabolic dependencies of the broad amino acid transporter SLC6A14
Source: Oncotarget. 2020 Dec 1;11(48):4490–503. doi: 10.18632/oncotarget.27758 (PMC7721610; doi:10.18632/oncotarget.27758)
Supplement: Supplementary file 1 [file oncotarget-11-4490-s001.pdf]

Exploiting the metabolic dependencies of the broad amino acid transporter SLC6A14

SUPPLEMENTARY MATERIALS

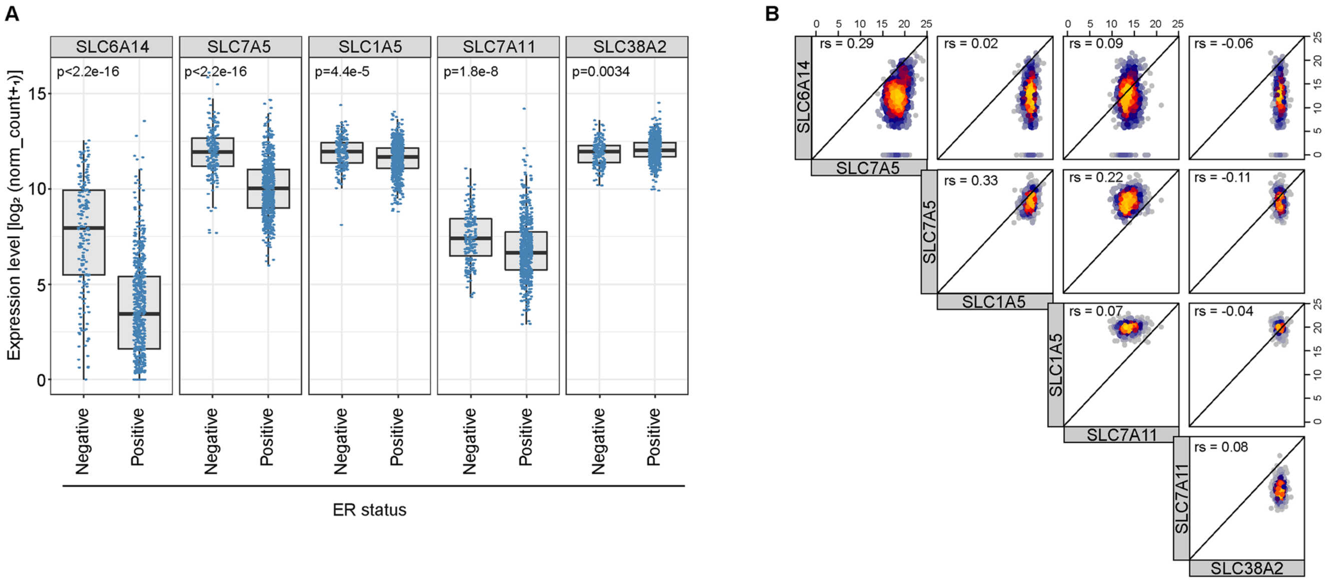

Supplementary Figure 1: (**A**) Expression levels of selected amino acid transporter genes in the BRCA dataset from TCGA, in which the information on the ER status was available ($n = 782$). Transcript levels are pan-cancer normalized. *P*-values were calculated using a Mann-Whitney *U* test. (**B**) Spearman correlation relative to the indicated targets in the BRCA gene expression dataset from TCGA ($n = 1218$).

A

Stable clone	Deletion (bp)	
	Allele I	Allele II
g1 #9 (KO1)	5	2
g1 #13 (KO2)	2	5
g2 #9 (KO3)	2	13

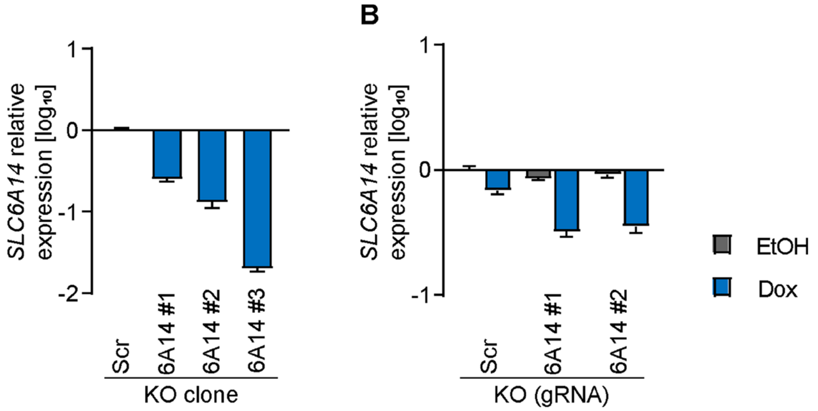

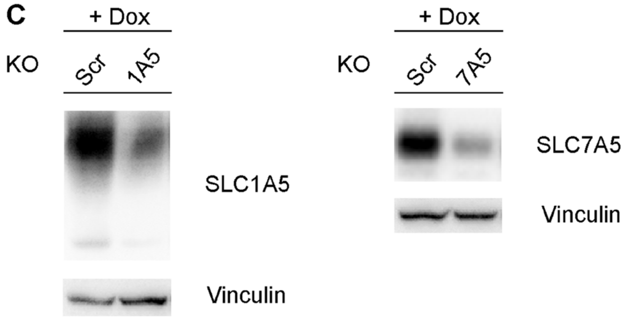

Supplementary Figure 2: (**A**) (Left) Table showing the gene editing event occurring in each selected SLC6A14 stable KO clone. Two different guide RNAs were used for generating the KO cell lines (g1 and g2). Indel formation was evaluated by Sanger sequencing. (Right) RT-qPCR of SLC6A14 mRNA levels in the corresponding SLC6A14 KO clones. Expression levels are normalized to those of a control scrambled KO clone. Data are representative of 2 independent experiments. Error bars indicate standard deviation of technical triplicates. (**B**) RT-qPCR of SLC6A14 mRNA levels in MDA-MB-468 inducible KO cell lines. Two different guide RNAs were used for generating the KO cell lines (g1 and g2). Cas9 expression was induced by treating cells stably expressing each gRNA with doxycycline. The KO efficiency was evaluated after 5 days and the expression levels were normalized to the control cell line (Scr KO) treated with EtOH. Data are representative of 3 independent experiments. Error bars indicate standard deviation of technical triplicates. (**C**) Immunoblot showing the protein levels of SLC1A5 and SLC7A5 in MDA-MB-468 inducible KO cell lines after 5 days of doxycycline treatment. Vinculin served as loading control. Data are representative of 3 independent experiments.

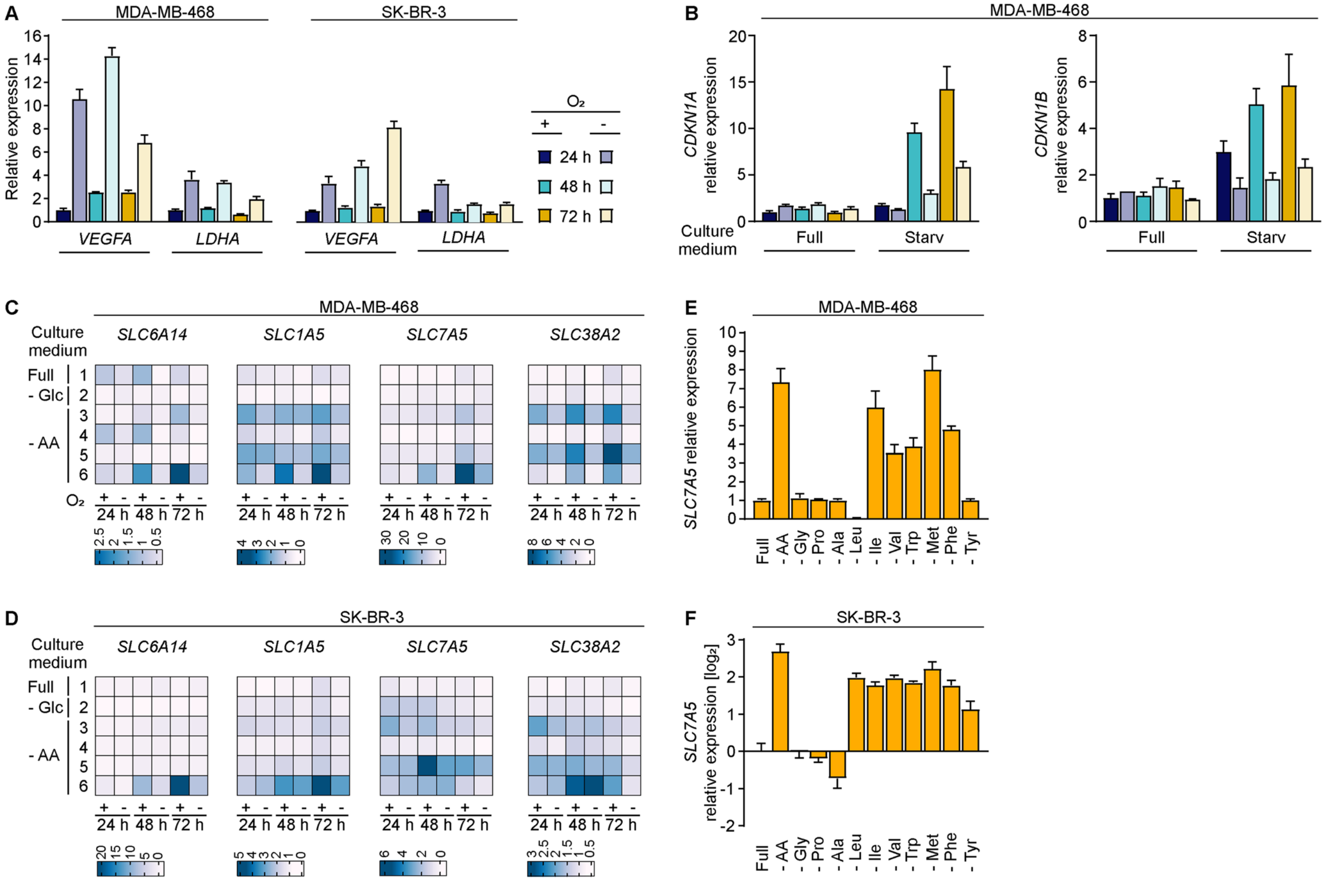

Supplementary Figure 3: (**A**) RT-qPCR of HIF-1α target genes in the indicated breast cancer cell line cultured in the complete medium upon hypoxia (+ O_2 = 20% oxygen; O_2 = 1% oxygen). Data are representative of 3 independent experiments. Error bars indicate standard deviation of technical triplicates. (**B**) RT-qPCR of CDKN1A and CDKN1B mRNA levels in MDA-MB-468 cells cultured under the indicated conditions (Full = complete medium; Starv = medium without aromatic and hydrophobic amino acids). Legend is indicated in Supplementary Figure 3A. Data are representative of 3 independent experiments. Error bars indicate standard deviation of technical triplicates. (**C**–**D**) Heat-maps summarizing RT-qPCR experiment results of the expression levels of SLC6A14, SLC1A5, SLC7A5 and SLC38A2 in the indicated breast cancer cell lines upon metabolic stress. Data show the average of independent replicates ($n \geq 2$). (**E**–**F**) RT-qPCR of SLC7A5 mRNA levels in MDA-MB-468 and SK-BR-3 breast cancer cell lines upon removal of the indicated amino acids for 72 h. Data are representative of 3 independent experiments. Error bars indicate standard deviation of technical triplicates.

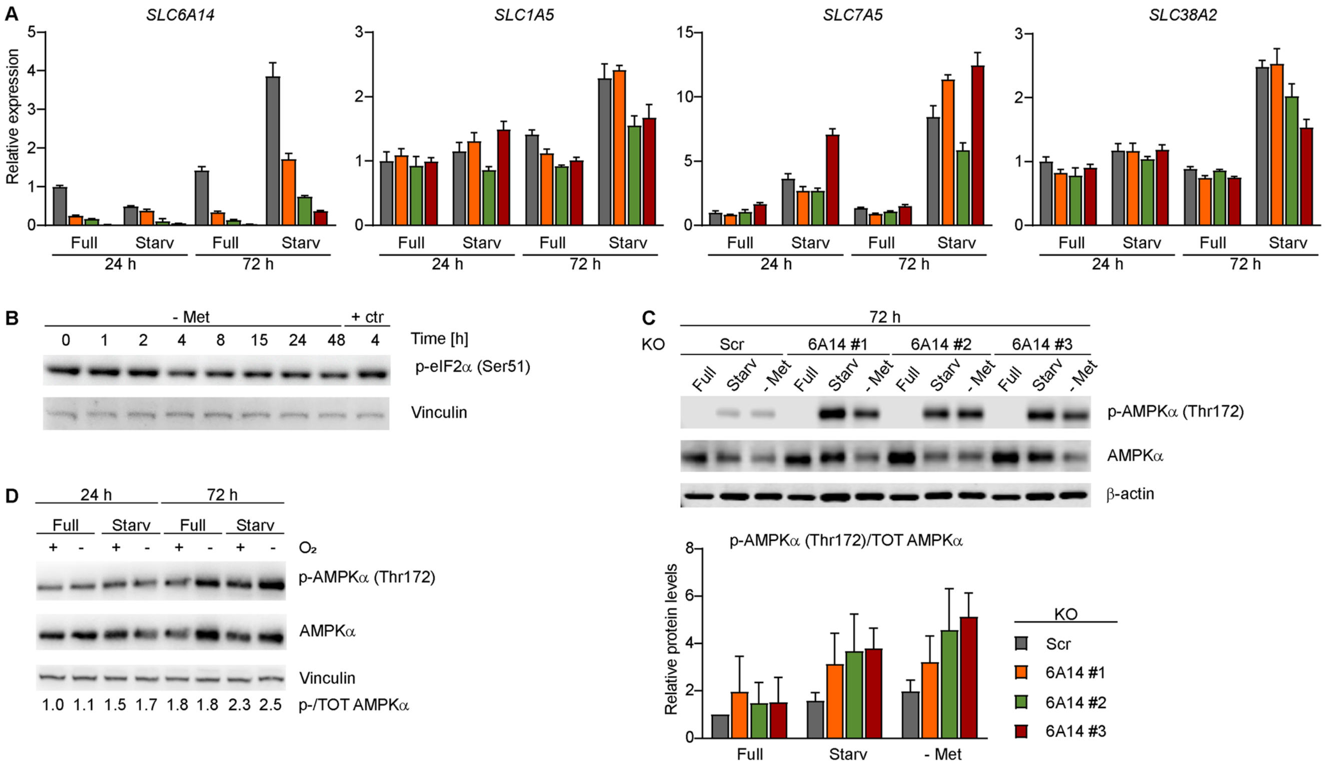

Supplementary Figure 4: (**A**) RT-qPCR showing mRNA levels of the indicated targets in stable MDA-MB-468 KO clones. Legend is indicated in Supplementary Figure 4C. Data are representative of 3 independent experiments. Error bars indicate standard deviation of technical triplicates. (**B**) Immunoblot showing time-dependent changes in the levels of phospho-eIF2α in MDA-MB-468 cells upon methionine starvation. Thapsigargin-treated cells (2 μM) were used as a positive control. Vinculin served as loading control. (**C**) Immunoblot of stable MDA-MB-468 KO clones. Cells were cultured for 72 h in the indicated media (Full = complete medium; Starv = medium without aromatic and hydrophobic amino acids; Met = medium without methionine). β-actin served as loading control. Immunoblot quantification of 3 independent experiments is shown below. Data represent mean + standard deviation. (**D**) Immunoblot showing AMPK activation in the MDA-MB-468 cell line cultured under the indicated conditions (+ O_2 = 20% oxygen; O_2 = 1% oxygen). Vinculin served as loading control. The normalized p-AMPKα/ AMPKα TOT ratio is shown below each band. Data are representative of 2 independent experiments.

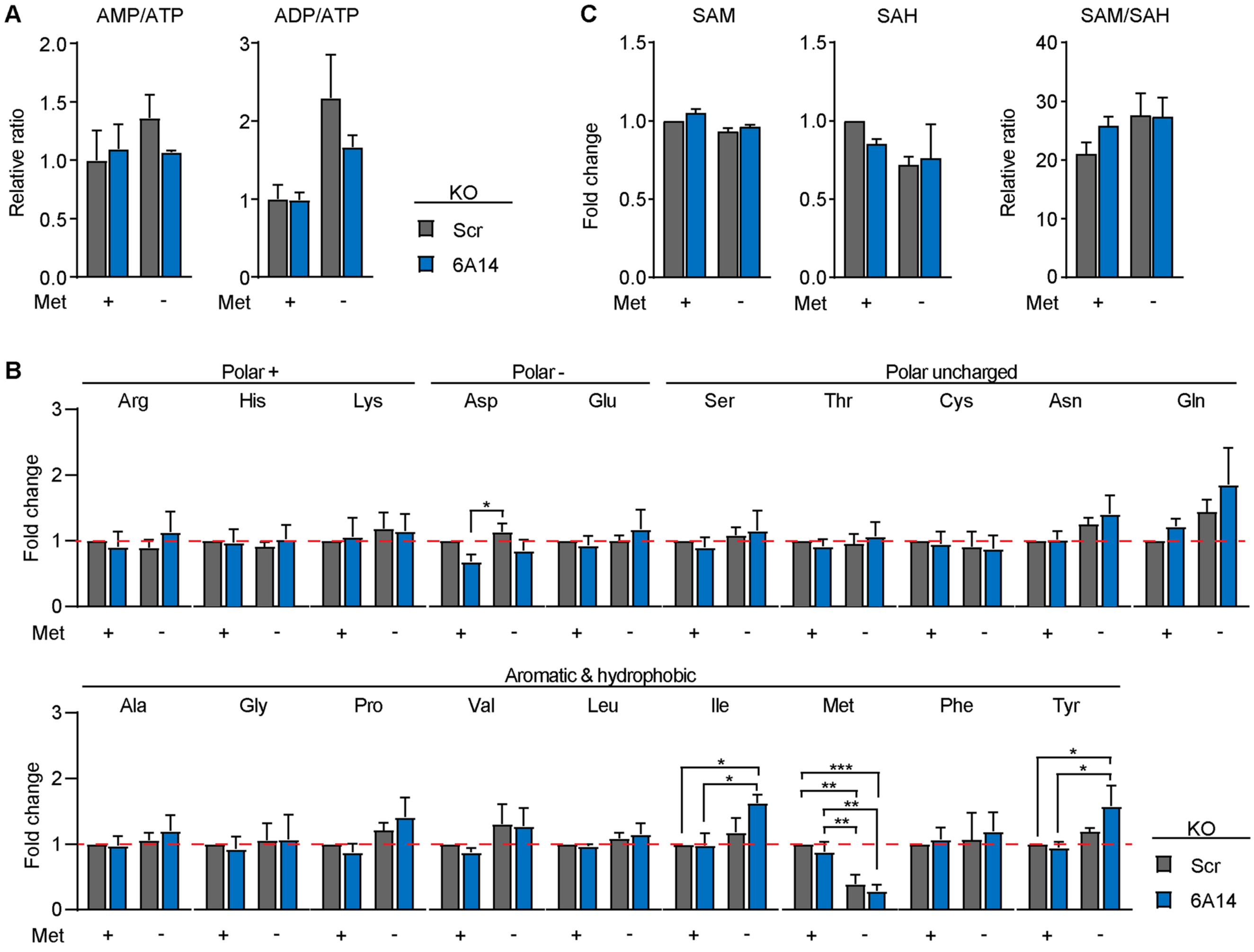

Supplementary Figure 5: (**A**) Relative AMP/ATP and ADP/ATP ratios in inducible MDA-MB-468 KO cell lines. Cells were induced with doxycycline for 4 days, plated and cultured for 24 h in the presence or absence of methionine before metabolite extractions. Values are normalized to Scr KO + Met condition. Data represent mean + standard error mean of 3 independent replicates. (**B**) Fold changes relative to Scr KO + Met condition of the indicated amino acid in inducible MDA-MB-468 KO cell lines treated as indicated in Supplementary Figure 5A. Error bars indicate standard deviation of 3 independent replicates. *P*-values are calculated using an ANOVA test followed by Tukey pairwise comparisons ($^{***}p \leq 0.001$; $^{**}p \leq 0.01$; $^{*}p \leq 0.05$). (**C**) (Left) Fold changes relative to Scr KO + Met condition of S-Adenosylmethionine (SAM) and S-adenosylhomocysteine (SAH) in inducible MDA-MB-468 KO cell lines. Error bars indicate standard error mean of 3 independent replicates. (Right) Relative SAM/SAH ratio. Data represent mean + standard error mean of 3 independent replicates. Cells were treated as indicated in Supplementary Figure 5A.

Supplementary Table 1: Formulation of RPMI medium used in this study

Metabolite name	Concentration (µM)	Order number (Sigma, unless specified)
**L-Amino acids**		
Alanine	430	A7627
Arginine	1149	A8094
Asparagine	378	A0884
Glycine	133	G7126
Proline	174	P0380
Serine	286	S4500
Aspartate	150	A9256
Cystine	208	C8755
Glutamate	136	G1251
Tyrosine	111	T3754
Histidine	97	H5659
Isoleucine	382	I2752
Leucine	382	L8000
Lysine	219	L5626
Methionine	101	M9625
Phenylalanine	91	P2126
Threonine	168	T8625
Tryptophan	25	T0254
Valine	171	V0500
Glutamine	2000	
**Vitamins**		
Biotin	0,819	
Choline	21,4	
Folate	2,3	
myo-Inositol	194,4	
Niacinamide	8,2	
p-Aminobenzoate	7,2	R7256
Pantothenate	0,524	
Pyridoxine	4,9	
Riboflavin	0,532	
Thiamine	2,9	
Vitamin B-12	0,00369	
**Inorganic Salts**		
$MgSO_4.7H_2O$	407	AppliChem A1037
KCl	5333	AppliChem A3582
NaCl	103448	S7653
$NaHCO_3$	23809	S5761
Na_2HPO_4	5634	S9390
$Ca(NO_3)_2.4H_2O$	424	C2786
**Other components**		
D-Glucose	25000	G7021
Glutathione (reduced)	3,2	G4251
HEPES	10000	Gibco 15630-080
Phenol Red	13	P5530
Sodium Pyruvate	1000	S8636

Supplementary Table 2: Oligonucleotides used for RT-qPCR

Target	Forward sequence (5′-3′)	Reverse sequence (5′-3′)
36B4	CCCATTCTATCATCAACGGGTACAA	CAGCAAGTGGGAAGGTGTAATCC
SLC6A14	TGGCTTGGCTCATAGTTGGA	TTGAAGCACCCTCCAGAGTT
SLC1A5	TCTCCTTGATCCTGGCTGTG	CCAGAGCGTCACCTTCTACA
SLC7A5	GTCCCTGTTCACATCCTCCA	TAGAGCAGCGTCATCACACA
SLC7A11	TCCGATCTTTGTTGCCCTCT	GTGCTTGCGGACATGAATCA
SLC38A2	CCGTCTGGCTGTGTTAATGG	ACTATGACGCCACCAACTGA
VEGFA	CCCACTGAGGAGTCCAACAT	TTTCTTGCGCTTTCGTTTTT
LDHA	TGGCAGCCTTTTCCTTAGAA	ACCAGCTTGGAGTTTGCAGT
CDKN1A	GGAAGACCATGTGGACCTGT	GGCGTTTGGAGTGGTAGAAA
CDKN1B	TAAGGAAGCGACCTGCAACC	TTGACGTCTTCTGAGGCCAG

Supplementary Table 3: Antibodies used for immunoblot (all obtained from Cell Signaling)

Target	Order number
β-Actin	3700
Vinculin	4650
Cleaved-PARP (Asp214)	5625
Caspase 3	9662
LAT1 (SLC7A5)	5347
ASCT2 (SLC1A5)	8057
eIF2α	9722
Phospho-eIF2α (Ser51)	3398
AMPKα	2532
Phospho-AMPKα (Thr172)	2535
4E-BP1	9452
Phospho-4E-BP1 (Ser65)	9451
P70S6K	2708
Phospho-p70S6K (Thr389)	9234

Supplementary Table 4A: gRNAs cloned into pLenti-Guide-Puro vector (including BsmBI cloning site, in bold) and used for KO cell line generation

Target	Forward sequence (5′-3′)	Reverse sequence (5′-3′)
Scrambled	**CACCG**ACGGAGGCTAAGCGTCGCAA	**AAAC**TTGCGACGCTTAGCCTCCGTC
SLC6A14 (#1)	**CACCG**ATCTATGATTGGATACGCAG	**AAAC**CTGCGTATCCAATCATAGATC
SLC6A14 (#2)	**CACCG**ATTGGATACGCAGTGGGATT	**AAAC**AATCCCACTGCGTATCCAATC
SLC1A5	**CACCG**CAGGCGGCTACTGCGGTTCC	**AAAC**GGAACCGCAGTAGCCGCCTGC
SLC7A5	**CACCGC**GTGGGGACCATTATCGGCT	**AAAC**AGCCGATAATGGTCCCCACGC

Supplementary Table 4B: sgRNAs used for transfection

Target	Forward sequence (5′-3′)
Scrambled	GACGTCTAGCTGGCTAGCAT
PRKAA1	GCGTGTCACCCAGAATGTAG
PRKAA2	GAAGATCGGACACTACGTGC
